# Supplementary material for: Exploring dopant effects in stannic oxide nanoparticles for CO2 electro-reduction to formate
Source: Nat Commun. 2022 Apr 22;13:2205. doi: 10.1038/s41467-022-29783-7 (PMC9033853; doi:10.1038/s41467-022-29783-7)
Supplement: Supplementary file 1 — Supplementary Information [file 41467_2022_29783_MOESM1_ESM.pdf]

Supplementary Information for

Exploring dopant effects in stannic oxide nanoparticles for CO_2 electro-reduction to formate

Young-Jin Ko,[a,†,] Jun-Yong Kim,[b,c,†] Woong Hee Lee,[a,†] Min Gyu Kim,[d] Tae-Yeon Seong,[c] Jongkil Park,[e] YeonJoo Jeong,[e] Byoung Koun Min,[a,f] Wook-Seong Lee,[b] Dong Ki Lee,[a,f,g,*] Hyung-Suk Oh [a,g,h,*]*

[a] Clean Energy Research Center, Korea Institute of Science and Technology (KIST), Hwarang-ro 14-gil 5, Seongbuk-gu, Seoul 02792, Republic of Korea

[b] Electronic Materials Research Center, Korea Institute of Science and Technology (KIST), Hwarang-ro 14-gil 5, Seongbuk-gu, Seoul 02792, Republic of Korea

[c] Department of Materials Science and Engineering, Korea University, Seoul 02841, Republic of Korea

[d] Beamline Research Division, Pohang Accelerator Laboratory (PAL), Pohang 37673, Republic of Korea

[e] Center for Neuromorphic Engineering, Korea Institute of Science and Technology (KIST), Hwarang-ro 14-gil 5, Seongbuk-gu, Seoul 02792, Republic of Korea

[f] Graduate School of Energy and Environment (Green School), Korea University, 145 Anam-ro, Seongbuk-gu, Seoul 02841, Republic of Korea

[g] Division of Energy and Environmental Technology, KIST school, Korea University of Science and Technology, Seoul 02792, Republic of Korea

[h] KIST-SKKU Carbon-Neutral Research Center, Sungkyunkwan University, 2066 Seobu-ro, Jangan-gu, Suwon 16419

† All authors contributed equally to this work.

***Corresponding Authors**

E-mail address:

091183@kist.re.kr (Y.-J. Ko), dnklee@kist.re.kr (D. K. Lee), hyung-suk.oh@kist.re.kr (H.-S. Oh)

Tel.: +82 (0)2 958 5292

Table of Contents

Contents		Page
○ Supplementary Note 1	…………………………………………………………	S3
> The overall synthesis mechanism of stannic oxide nanoparticles.		
○ Supplementary Note 2	…………………………………………………………	S4
> The reason of different electrodes for OER in neutral and alkaline electrolytes.		
○ Supplementary Figures	…………………………………………………………	S5
□ Supplementary Figures 1–17		
○ Supplementary Tables	…………………………………………………………	S22
□ Supplementary Tables 1–5		
○ References	…………………………………………………………	S28

Supplementary Note 1. The overall synthesis mechanism of stannic oxide nanoparticles.

(1) Formation of metal-surfactant complex: Tin(IV) chloride ($SnCl_4$) reacts rapidly with ethanol to form tin ethobutoxide [$Sn(OC_2H_5)_4$], as indicated by the following reaction.

$$SnCl_4 + 4C_2H_5OH \rightarrow Sn(OC_2H_5)_4 + 4\mathrm{HCl}$$

The ethanol solution becomes acidic during the formation of the complex, and the hydrolysis reaction is significantly slowed down at low pH.

(2) Hydrolysis and condensation: In order to accelerate the hydroxide reaction, ammonia hydroxide (NH_4OH) is added to increase the pH. White precipitation is observed at higher pH, which is clear evidence of the hydrolysis and condensation phenomenon.

(3) Formation of micelles-like surfactant template with SnO_2 phase: The micelles of the tetradecylamine (TDA) shell on to metal oxide are in an intermediate phase. The related structures were obtained through an alkylamine (TDA)-tin ethobutoxide. TDA further induce the binding between metal oxide nanoparticles and the amine functional group by hydrogen bonding. Finally, the micelles consist of a core of hydrophobic alkyl moiety and a shell of hydrophilic amine-metal oxide complex.

(4) Hydrothermal treatment for crystallization: Hydrothermal treatment was performed to increase the crystallinity of oxide catalysts, which improved the thermal and chemical stability.

Supplementary Note 2. The reason for the use of different OER electrodes in neutral and alkaline electrolytes.

Fe-Ni foam exhibits excellent OER performance under alkaline conditions. At the same time, it is cheaper than an iridium catalyst. However, it slowly dissolves in neutral conditions, leading to the degradation of the OER performance of the electrode. Furthermore, the dissolved Ni ion can cross over to the cathode, reducing the FE for CO_2RR and inducing the hydrogen evolution reaction (HER). Therefore, IrO_2/Pt coated Ti-foam electrodes were used for the OER in neutral media for a stable CO_2RR operation.

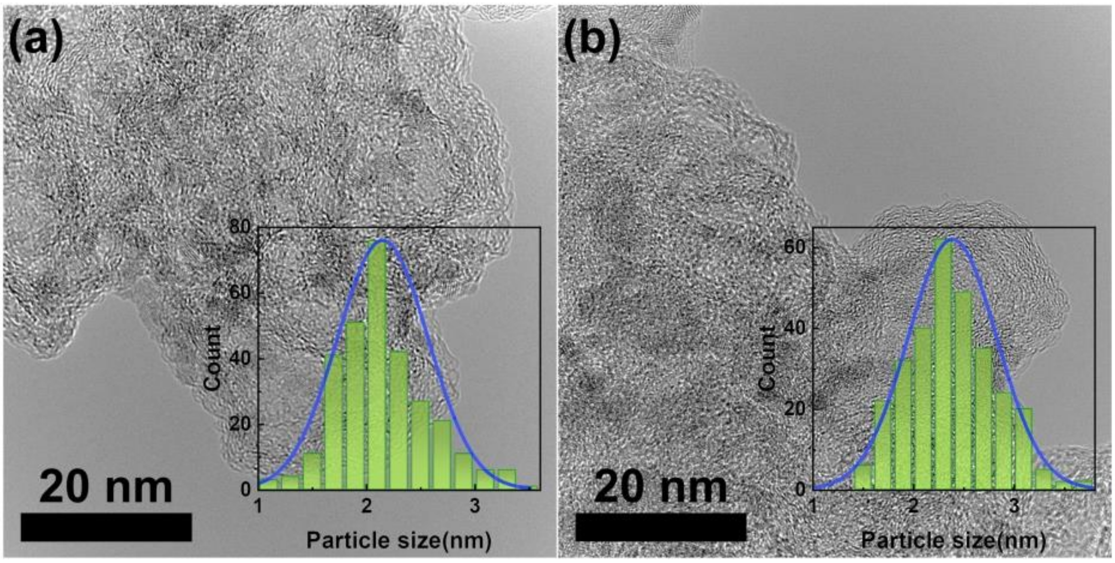

Supplementary Figure 1. HR-TEM images of prepared Sn-based electrocatalysts. (a) ATO/C and (b) ITO/C catalysts (Inset: the particle size distributions; average particle sizes and standard deviations were fitted with a Gaussian function).

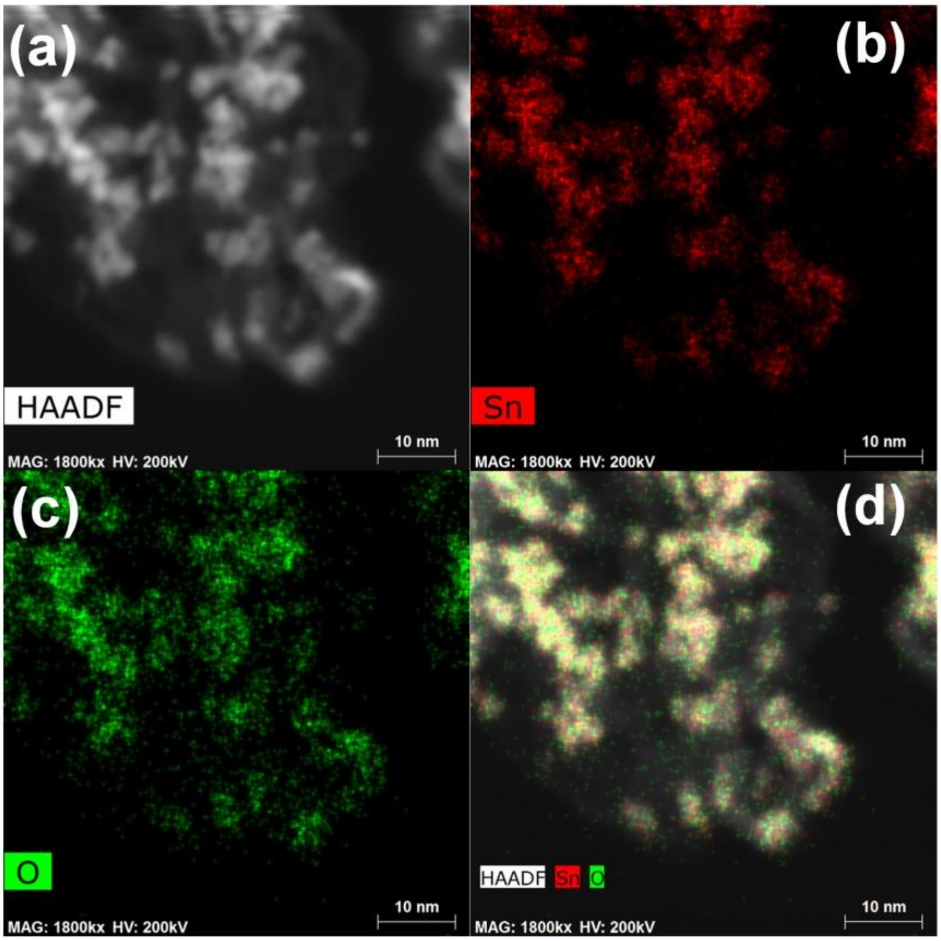

Supplementary Figure 2. STEM images of SnO_2/C. (a) HAADF-STEM image and its energy dispersive X-ray spectroscopy (EDS) elemental mapping images of (b) Sn (red), (c) O (green) and (d) layered image combining all 3 maps for SnO_2/C. The signal collecting time was 5 minutes.

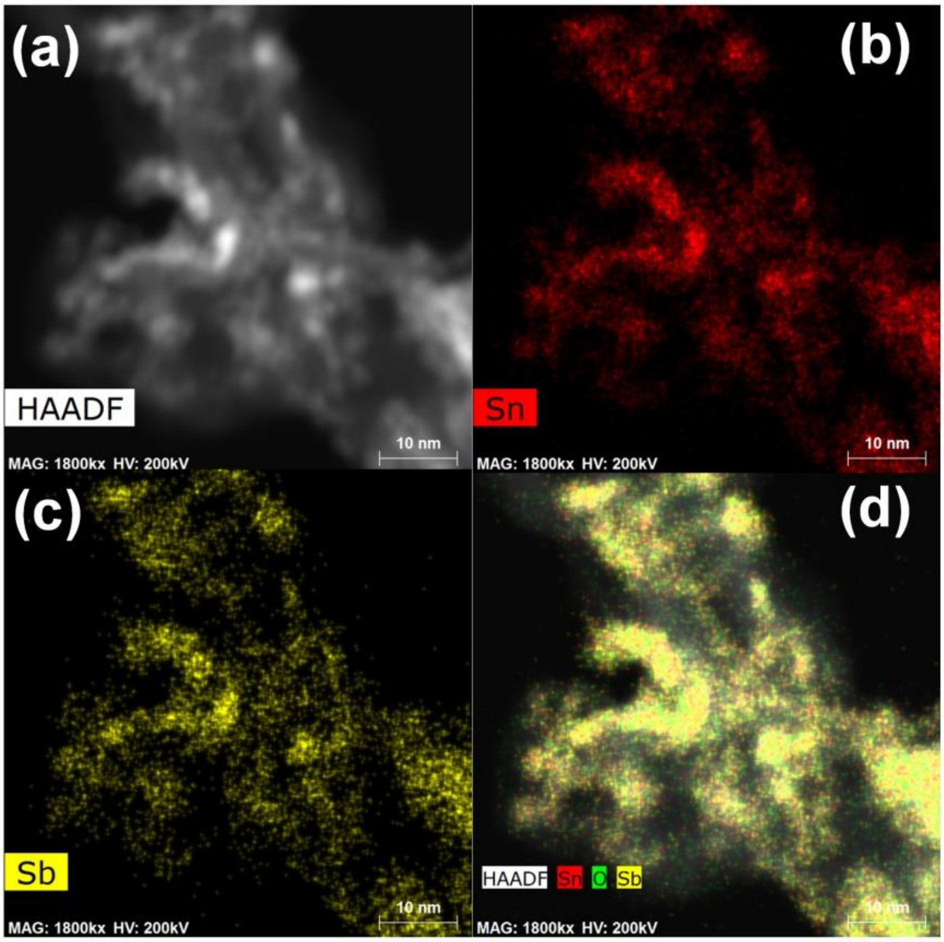

Supplementary Figure 3. STEM images of ATO/C. (a) HAADF-STEM image and its energy dispersive X-ray spectroscopy (EDS) elemental mapping images of (b) Sn (red), (c) Sb (yellow) and (d) layered image combining all maps for ATO/C. The signal collecting time was 5 minutes.

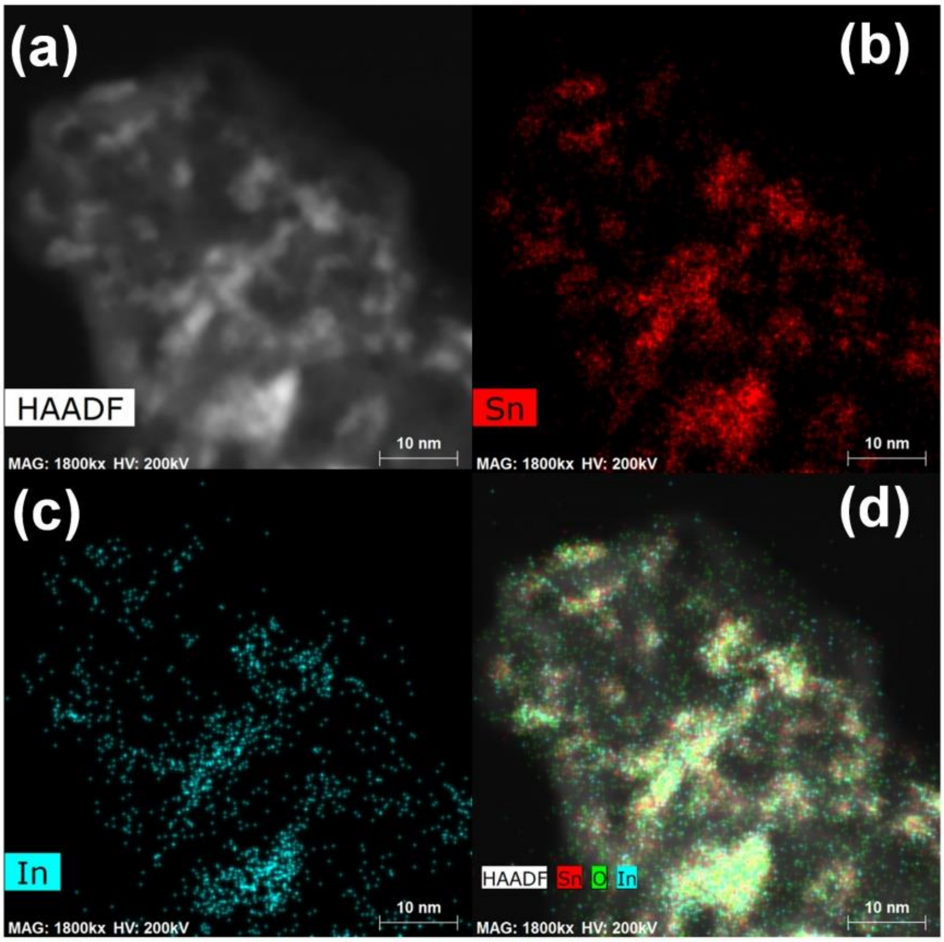

Supplementary Figure 4. STEM images of ITO/C. (a) HAADF-STEM image and its energy dispersive X-ray spectroscopy (EDS) elemental mapping images of (b) Sn (red), (c) In (cyan) and (d) layered image combining all maps for ITO/C. The signal collecting time was 5 minutes.

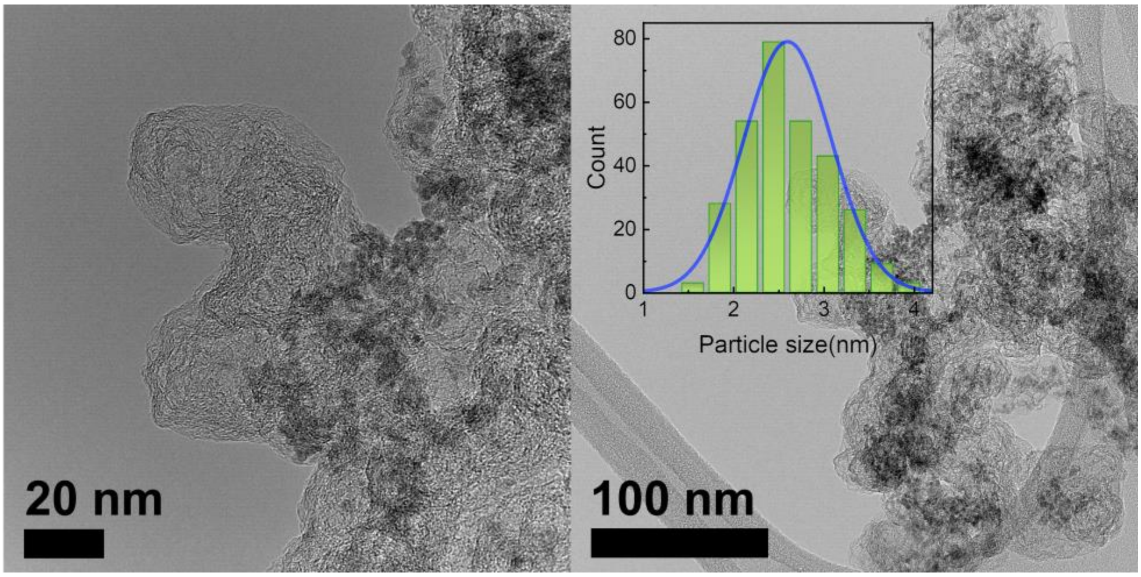

Supplementary Figure 5. HR-TEM image of SnO_2/C catalysts without TDA surfactant. Inset: the particle size distributions; average particle sizes and standard deviations were fitted with a Gaussian function).

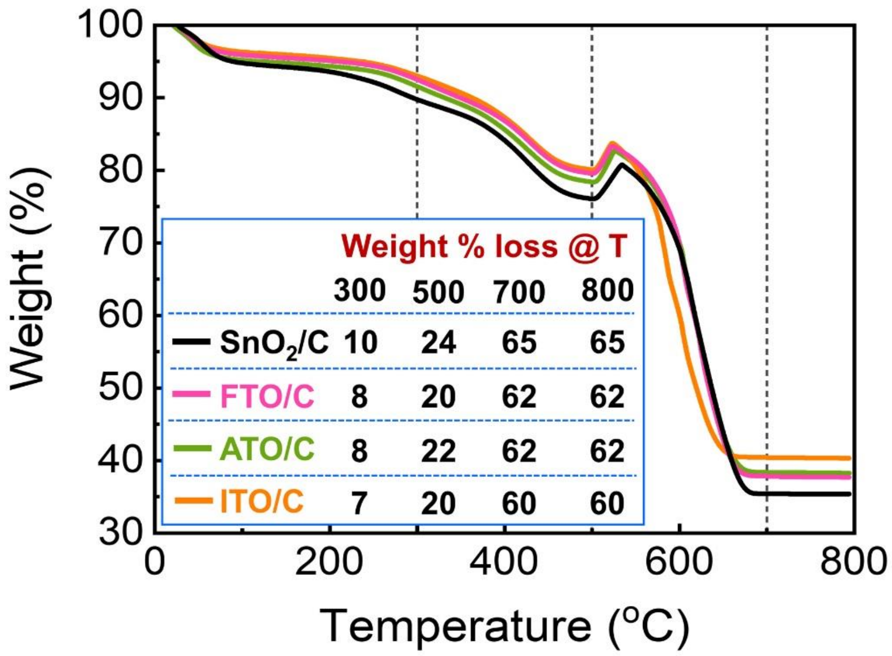

Supplementary Figure 6. Thermogravimetric analysis (TGA) curves of Sn-based catalysts. Heating rate was 10 °C min^{-1}. The analysis was performed in a N_2 atmosphere up to 500 °C, and O_2 gas was injected thereafter.

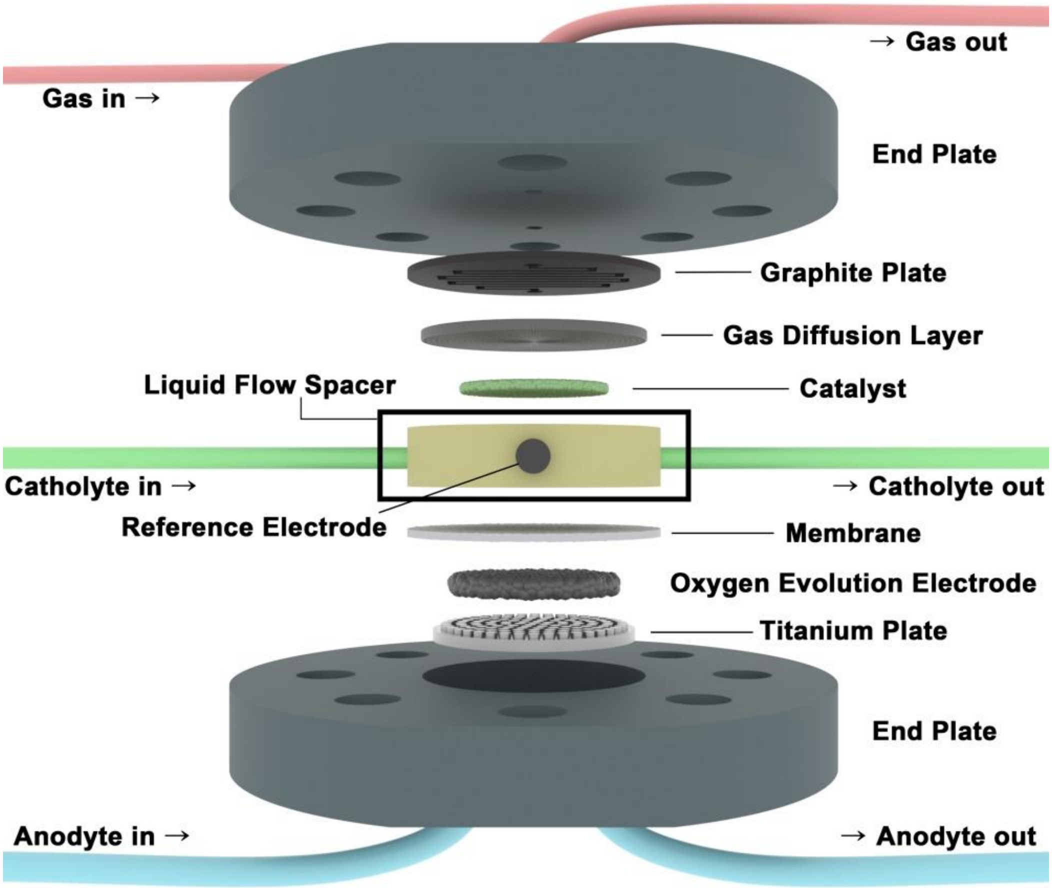

Supplementary Figure 7. Schematic illustration of the components for the flow-type CO_2 electrolyzer. The electrode area was 2 cm^2 and the loading of SnO_2 was fixed at 0.5 mg cm^{-2}.

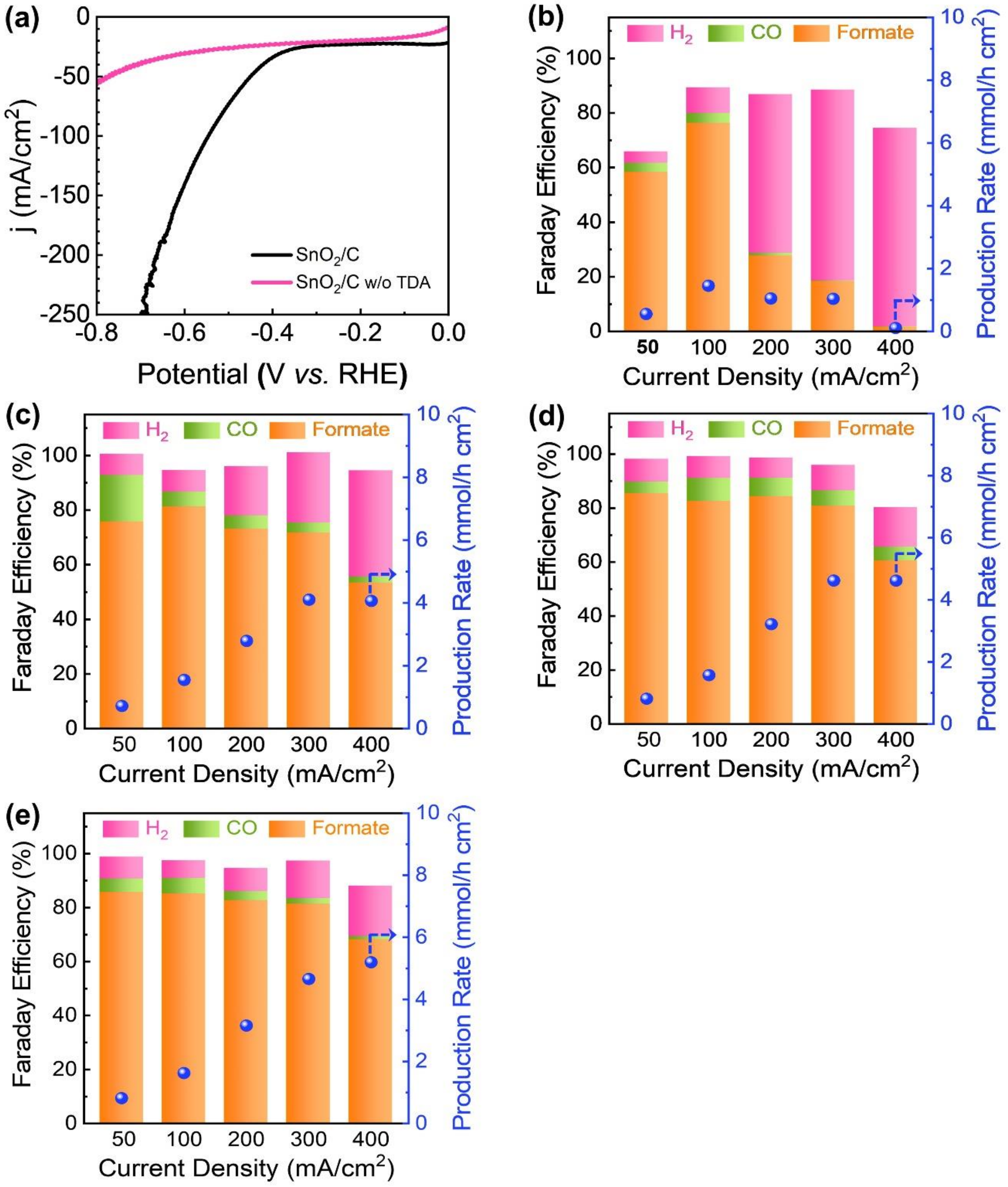

Supplementary Figure 8. Catalytic activity of Sn-based electrocatalysts for CO_2RR. (a) LSV curve for SnO_2/C catalysts with and without TDA surfactant and FE of products and production rates of formate for (b) SnO_2/C without TDA surfactant, (c) SnO_2/C with TDA surfactant, (d) ATO/C and (e) ITO/C catalysts at each given current densities. Reaction conditions: 1 M KOH solution (alkaline condition).

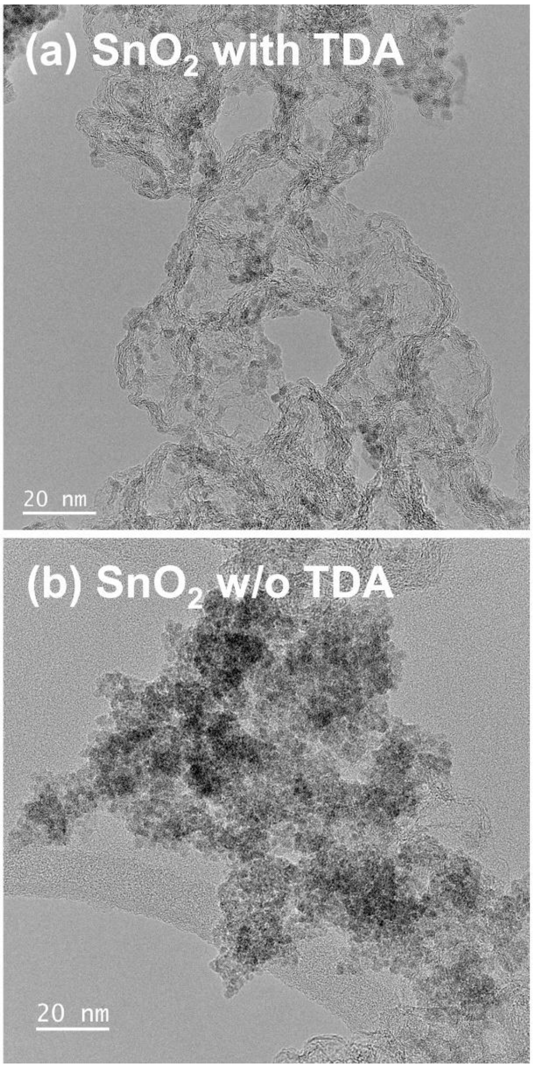

Supplementary Figure 9. HR-TEM images of Sn-based electrocatalysts. SnO_2/C (a) with and (b) without TDA surfactant after CO_2RR.

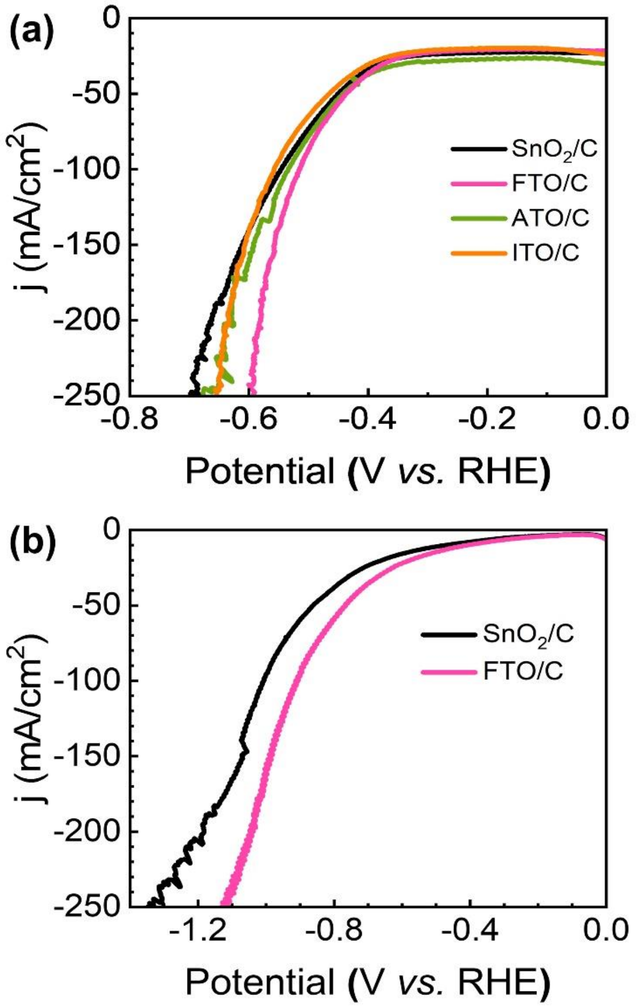

Supplementary Figure 10. LSV curves for SnO_2-based catalysts with different electrolyte conditions. (a) 1 M KOH solution and (b) 1 M $KHCO_3$ solution.

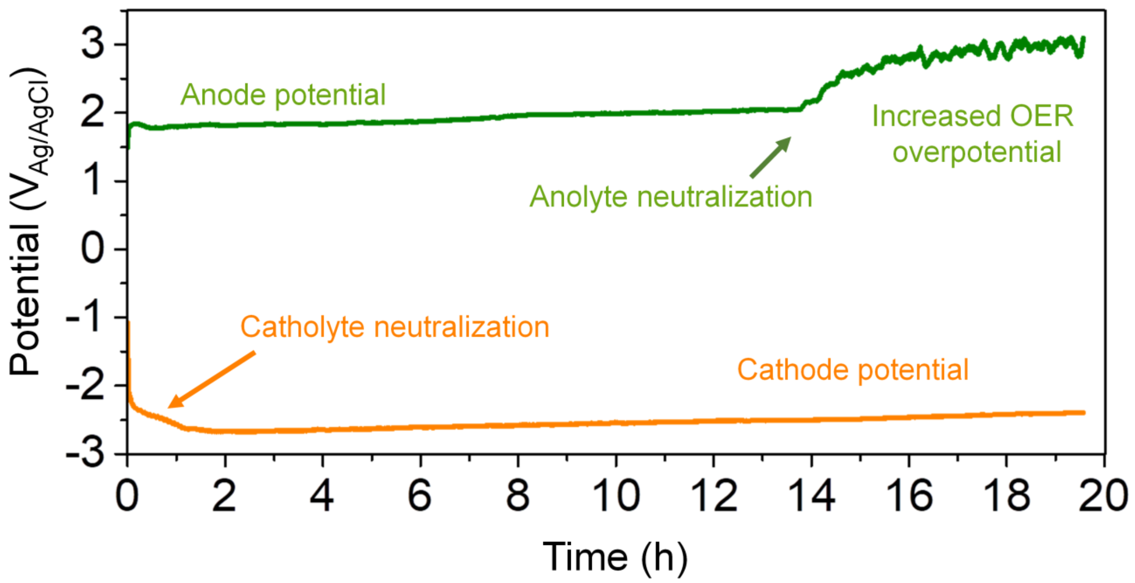

Supplementary Figure 11. Electrochemical stability test result in 1 M KOH electrolyte. The anode and cathode potentials (vs. Ag/AgCl reference electrode) without iR-correction at a current density of 100 mA cm^{-2} in 1 M KOH solution in the flow-type CO_2 electrolyzer.

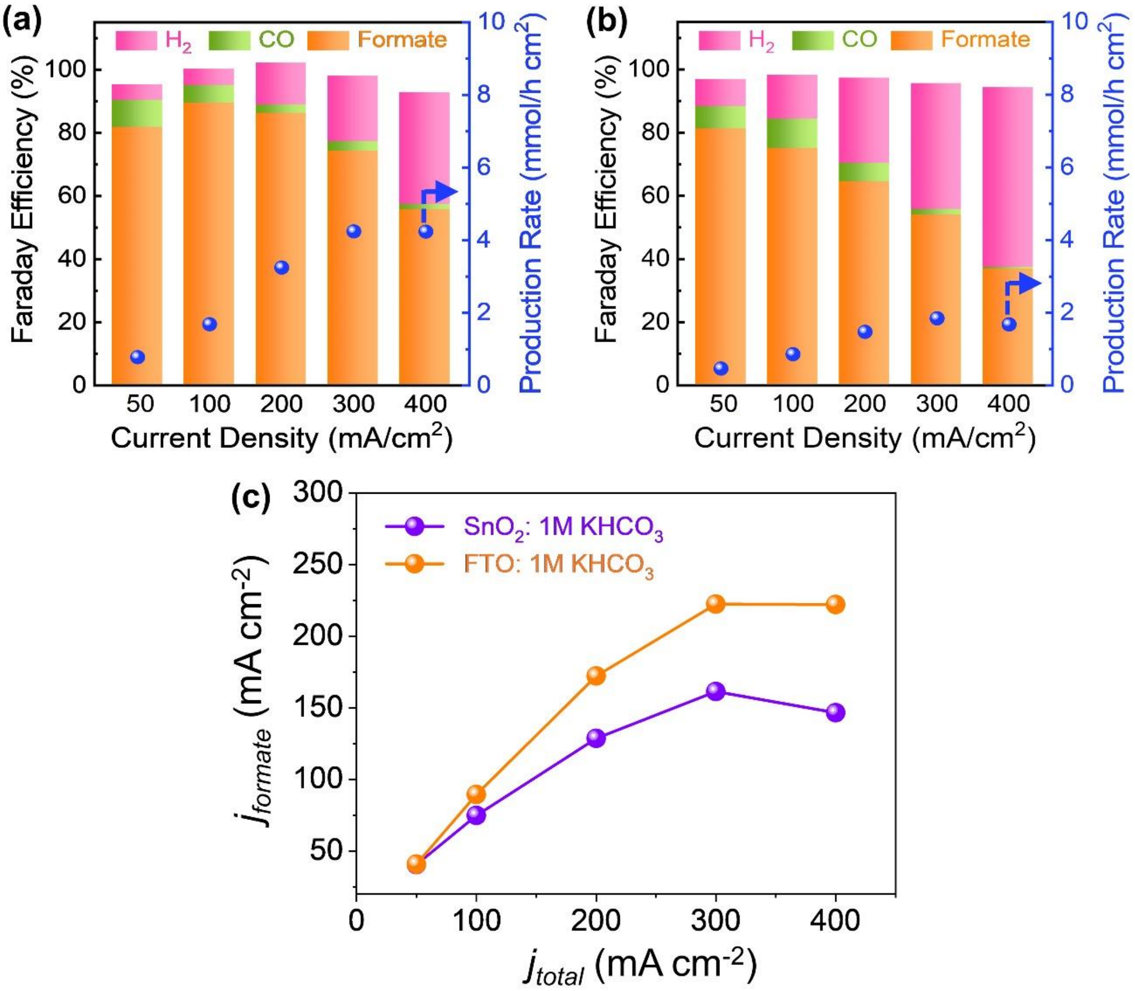

Supplementary Figure 12. Electrochemical performance for CO_2 conversion to formate in 1 M $KHCO_3$. FE of products and production rates of formate for (a) SnO_2/C and (b) FTO/C catalysts under 1 M $KHCO_3$ solution at each given current densities. (c) Partial current densities of formate for CO_2RR in the current density range of 50-400 mA cm^{-2} over SnO_2/C and FTO/C.

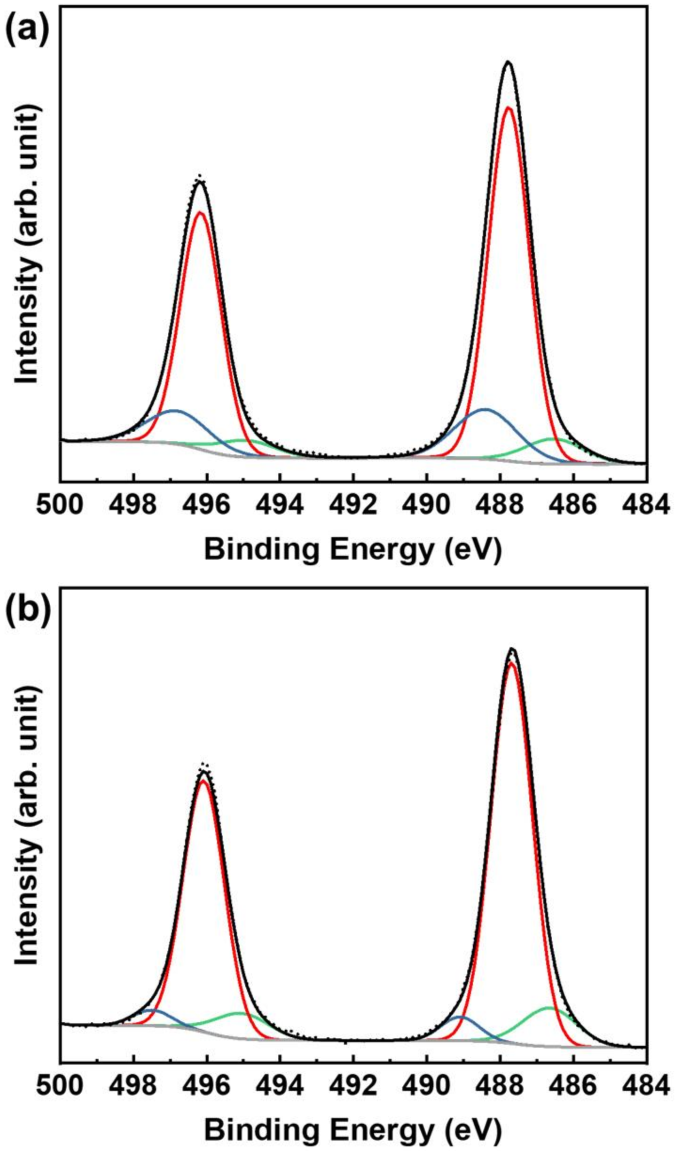

Supplementary Figure 13. XPS spectra of Sn 3*d* on FTO/C. (a) Before and (b) after electrochemical stability tests.

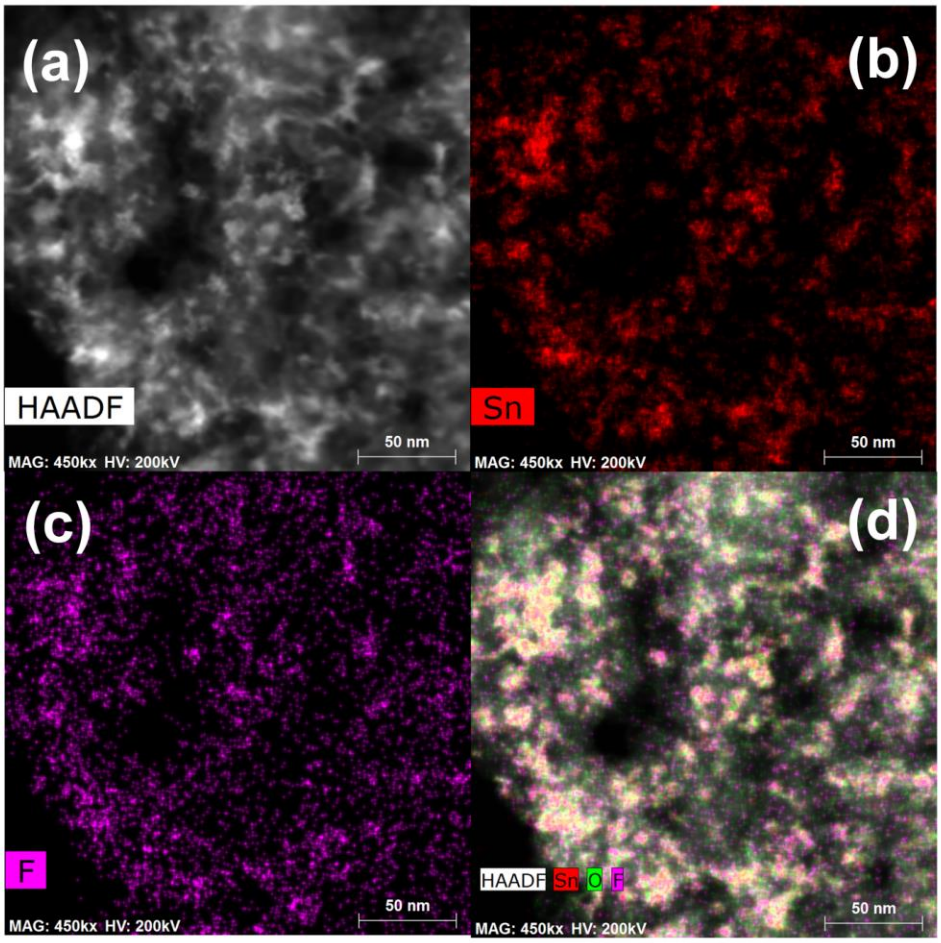

Supplementary Figure 14. STEM images of FTO/C after electrochemical stability test. (a) HAADF-STEM image and its energy dispersive X-ray spectroscopy (EDS) mapping images of (b) Sn (red), (c) F (magenta) and (d) layered image combining all 3 maps for FTO/C after stability test. The signal collecting time was 5 minutes.

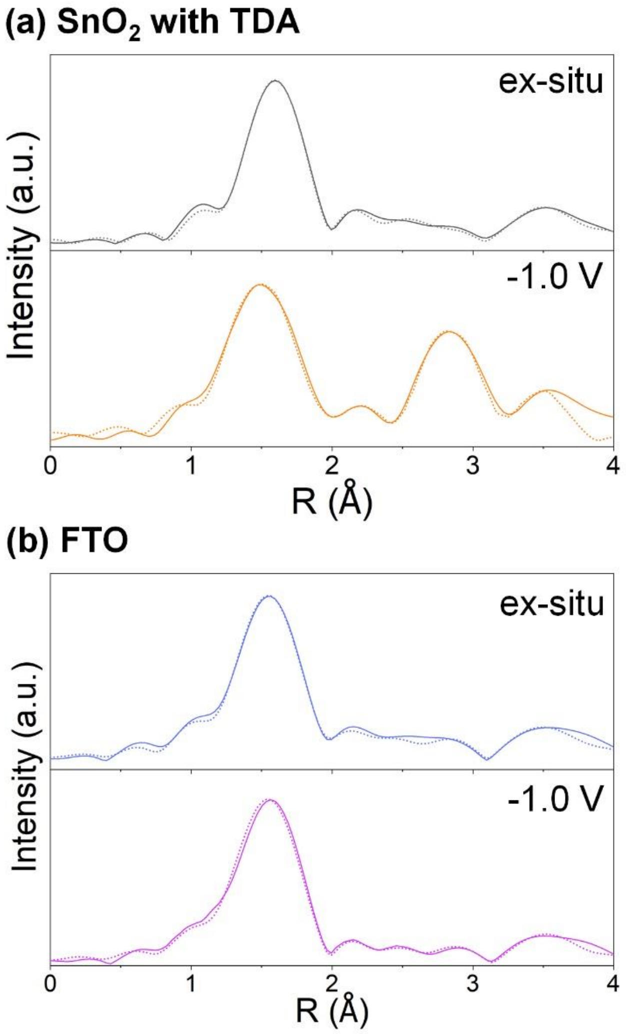

Supplementary Figure 15. Fourier transforms of k^3-weighted Sn L_{III}-edge EXAFS. (a) SnO_2 and (b) FTO catalyst obtained by *in-situ/operando* hard-XAS analysis

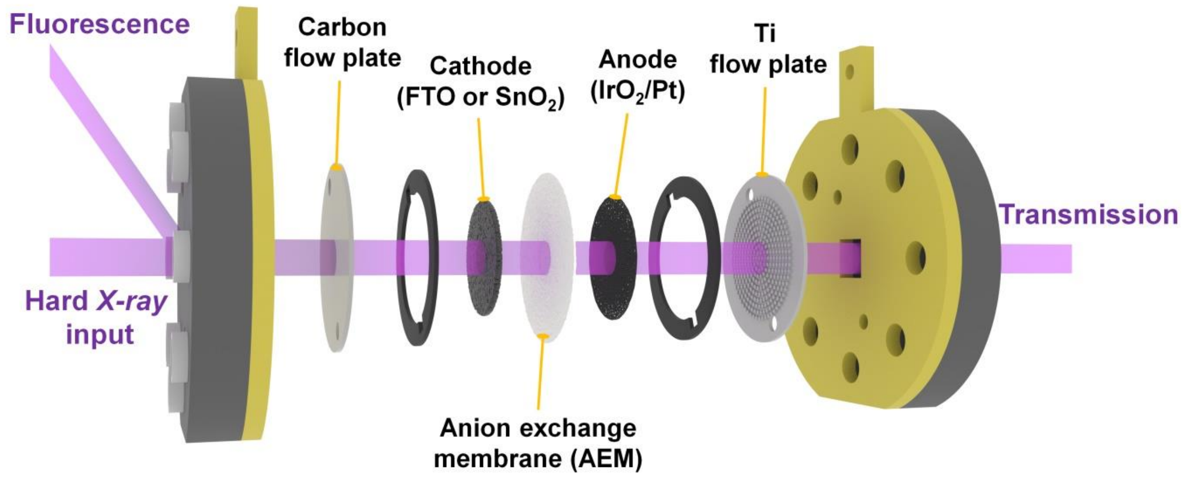

Supplementary Figure 16. System of *in-situ/operando* hard-XAS analysis with the homemade electrochemical zero-gap device. The operational conditions were the same as in the single-cell tests, and 1 M $KHCO_3$ was used as the catholyte and anolyte. Electrodes geometric area was 10 cm^2. 1 cm^2 hole was made in the anode and cathode bipolar plate and covered with Kapton film to allow passage of the X-rays.

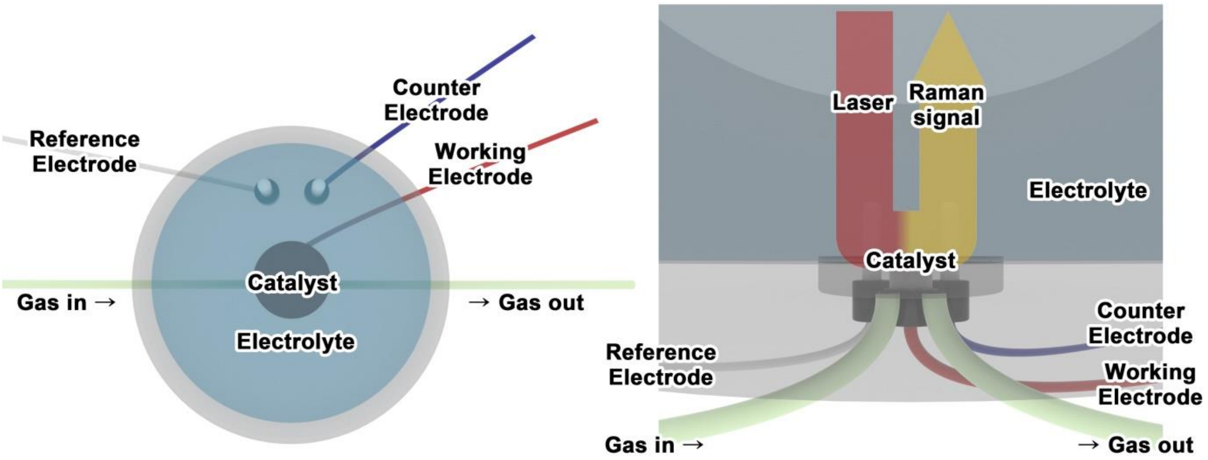

Supplementary Figure 17. Scheme of electrochemical flow cell for *in-situ/operando* surface-enhanced Raman spectroscopy (SERS). The homemade electrochemical flow-type device with reference electrode (Ag/AgCl), counter electrode (Pt wire), and working electrode.

Supplementary Table 1. Comparisons of CO_2RR to formate electrocatalytic activity and stability of Sn-based electrodes.

Cell type	Sn catalyst	Cathode feed	Maximum Formate F.E. (%)	Maximum Formate partial current density (mA cm^{-2})	Stability test (h)	Reference
Flow cell	**FTO/C**	**1 M KOH + CO_2 gas**<br>**1 M $KHCO_3$ + CO_2 gas**	**95.5**	**323**	**168**	**This work**
	Sn	1 M $KHCO_3$ + CO_2 gas	85	85	-	Jiao et al[1]
	Sn_3O_4 NS	1 M KOH + CO_2 gas	83	465	3	Zhu et al[2]
	SnO_2 nanoparticle	0.5 M Na_2CO_3 + CO_2 gas	72	277	-	Brushett et al[3]
	SnO_2 nanorod	1 M KOH + CO_2 gas	87	383	-	Lin et al[4]
	SnO_2/C	0.4M K_2SO_4+ CO_2 gas	90	450	11	Neyerlin et al[5]
	SnO_2 nanoparticle	1 M KOH + CO_2 gas	64	93	-	Ajayan et al[6]
	SnO_2 nanoparticle	0.1 M $KHCO_3$ + CO_2 gas	90	180	5	Klemm et al[7]
	Cu-Sn GDL	1 M KOH + CO_2 gas	78	88	4	Ge et al[8]
	SnO2 nanosheet	1 M KOH + CO_2 gas	94	471	3	Lu et al[8]
	Dense tips Sn	1 M $KHCO_3$ + CO_2 gas	68	55	72	Lee et al[9]
	Cu-Sn/SnO_x	1 M KOH + CO_2 gas	98	407	40	Wang et al[10]

H-cell	Cu@Sn,	0.5 M $KHCO_3$	100	16.52	20	Qiao et al[11]
	Cu supported dendritic Sn	0.5 M $NaHCO_3$	67.3	9.42	20	Zhang et al[12]
	Reduced SnO_2 porous nanowire	0.1 M $KHCO_3$	80	10	15	Spurgeon et al[13]
	Hierarchical mesoporous SnO_2 nanosheets	0.5 M $NaHCO_3$	83	16	12	Li et al[14]
	SnS/Au	0.1 M $KHCO_3$	93.3	55	40	Sargent et al[15]
	Sn/SnS_2	0.5 M $KHCO_3$	85	9.98	13	Zhang et al[16]
	Bi-Sn	0.1 M $KHCO_3$	90	18	12	Zhang et al[17]
	Bi-Sn	0.5 M $KHCO_3$	96	45	100	Chen et al[18]
	N-SnO_2 NS	0.1 M $KHCO_3$	83	16	10	Hou et al[19]

Supplementary Table 2. DFT calculated adsorption energy (*ΔE*), zero-point energy (*ZPE*), and entropy (*S*) at 298 *K* and 1 atm of CO_2 and HCOO adsorbate on the slab surface.

	**Adsorbed surface**	***ΔE* (eV)**	***ZPE* (eV)**	***TS* (eV)**
**CO_2***	SnO_2(110)	-0.347	0.312	0.020
	SnO_2(110), -1.37% strained	-0.354	0.320	0.025
	FTO(110)	-0.091	0.309	0.037
	FTO(110), -0.33% strained	0.112	0.308	0.033
**HCOO***	SnO_2(110)	-2.649	0.686	0.169
	SnO_2(110), -1.37% strained	-2.692	0.689	0.174
	FTO(110)	-3.484	0.666	0.193
	FTO(110), -0.33% strained	-3.235	0.661	0.189

Supplementary Table 3. EXAFS fitting results of SnO_2 and FTO catalysts. The multi-shell fitting results (Δk = 2 ~ 12 $Å^{-1}$) of the experimental EXAFS spectrum.

Catalyst	Condition	Sn-Sn bond	Sn-F bond	Sn-O bond
SnO_2	ex-situ	3.2595	-	2.0762
	-1.0 V	3.2063	-	2.0541
FTO	ex-situ	-	2.0359	2.0975
	-1.0 V	-	2.0305	2.0905

Supplementary Table 4. Summary of electrocatalysts, electrolyte and formate electrocatalytic activity for all device tests.

**Cathode**	**Anode**	**Electrolyte**	**Maximum Formate F.E (%)**	**Maximum Formate partial current density (mA cm^{-2})**
SnO_2/C w/o TDA	Fe-Ni foam	1 M KOH	76	76
SnO_2/C with TDA	Fe-Ni foam	1 M KOH	80	215
	IrO_2/Pt coated Ti-foam	1 M $KHCO_3$	75	161
FTO/C with TDA	Fe-Ni foam	1 M KOH	95	330
	IrO_2/Pt coated Ti-foam	1 M $KHCO_3$	89	222
ATO/C with TDA	Fe-Ni foam	1 M KOH	80	242
ITO/C with TDA	Fe-Ni foam	1 M KOH	85	272

Supplementary Table 5. Zero-point energy correction and entropy contribution of H_2, CO_2, and HCOOH gas at 298 *K* and 1 atm.

	***ZPE* (eV)**	***TS* (eV)**
**H_2**	0.276	0.441
**CO_2**	0.308	0.656
**HCOOH**	0.854	1.071

Supplementary References

1. Luc, W.; Ko, B. H.; Kattel, S.; Li, S.; Su, D.; Chen, J. G.; Jiao, F., SO_2-Induced Selectivity Change in CO_2 Electroreduction. *Journal of the American Chemical Society* **2019,** *141* (25), 9902-9909.
2. Liu, L.-X.; Zhou, Y.; Chang, Y.-C.; Zhang, J.-R.; Jiang, L.-P.; Zhu, W.; Lin, Y., Tuning Sn_3O_4 for CO_2 reduction to formate with ultra-high current density. *Nano Energy* **2020,** *77*, 105296.
3. Sen, S.; Brown, S. M.; Leonard, M.; Brushett, F. R., Electroreduction of carbon dioxide to formate at high current densities using tin and tin oxide gas diffusion electrodes. *Journal of Applied Electrochemistry* **2019,** *49* (9), 917-928.
4. Qian, Y.; Liu, Y.; Tang, H.; Lin, B.-L., Highly efficient electroreduction of CO_2 to formate by nanorod@2D nanosheets SnO. *Journal of CO_2 Utilization* **2020,** *42*, 101287.
5. Chen, Y.; Vise, A.; Klein, W. E.; Cetinbas, F. C.; Myers, D. J.; Smith, W. A.; Deutsch, T. G.; Neyerlin, K. C., A Robust, Scalable Platform for the Electrochemical Conversion of CO_2 to Formate: Identifying Pathways to Higher Energy Efficiencies. *ACS Energy Letters* **2020,** *5* (6), 1825-1833.
6. Liang, C.; Kim, B.; Yang, S.; Yang, L.; Francisco Woellner, C.; Li, Z.; Vajtai, R.; Yang, W.; Wu, J.; Kenis, P. J. A.; Ajayan, Pulickel M., High efficiency electrochemical reduction of CO_2 beyond the two-electron transfer pathway on grain boundary rich ultra-small SnO_2 nanoparticles. *Journal of Materials Chemistry A* **2018,** *6* (22), 10313-10319.
7. Kopljar, D.; Inan, A.; Vindayer, P.; Wagner, N.; Klemm, E. J. J. o. A. E., Electrochemical reduction of CO_2 to formate at high current density using gas diffusion electrodes. **2014,** *44* (10), 1107-1116.
8. Rabiee, H.; Zhang, X.; Ge, L.; Hu, S.; Li, M.; Smart, S.; Zhu, Z.; Yuan, Z., Tuning the Product Selectivity of the Cu Hollow Fiber Gas Diffusion Electrode for Efficient CO_2 Reduction to Formate by Controlled Surface Sn Electrodeposition. *ACS Applied Materials & Interfaces* **2020,** *12* (19), 21670-21681.
9. Lim, J.; Kang, P. W.; Jeon, S. S.; Lee, H., Electrochemically deposited Sn catalysts with dense tips on a gas diffusion electrode for electrochemical CO_2 reduction. *Journal of Materials Chemistry A* **2020,** *8* (18), 9032-9038.
10. Ye, K.; Zhou, Z.; Shao, J.; Lin, L.; Gao, D.; Ta, N.; Si, R.; Wang, G.; Bao, X., In Situ Reconstruction of a Hierarchical Sn-Cu/SnO_x Core/Shell Catalyst for High-Performance CO_2 Electroreduction. *Angewandte Chemie International Edition* **2020,** *59* (12), 4814-4821.
11. Hou, X.; Cai, Y.; Zhang, D.; Li, L.; Zhang, X.; Zhu, Z.; Peng, L.; Liu, Y.; Qiao, J. J. J. o. M. C. A., 3D core–shell porous-structured Cu@ Sn hybrid electrodes with unprecedented selective CO_2-into-formate electroreduction achieving 100%. **2019,** *7* (7), 3197-3205.
12. Zhang, Y.; Zhang, X.; Bond, A. M.; Zhang, J. J. P. C. C. P., Identification of a new substrate effect that enhances the electrocatalytic activity of dendritic tin in CO_2 reduction. **2018,** *20* (8), 5936-5941.
13. Kumar, B.; Atla, V.; Brian, J. P.; Kumari, S.; Nguyen, T. Q.; Sunkara, M.; Spurgeon, J. M. J. A. C. I. E., Reduced SnO2 porous nanowires with a high density of grain boundaries as catalysts for efficient electrochemical CO_2-into-HCOOH conversion. **2017,** *56* (13), 3645-3649.
14. Han, N.; Wang, Y.; Deng, J.; Zhou, J.; Wu, Y.; Yang, H.; Ding, P.; Li, Y. J. J. o. M. C. A., Self-templated synthesis of hierarchical mesoporous SnO_2 nanosheets for selective CO_2 reduction. **2019,** *7* (3), 1267-1272.
15. Zheng, X.; De Luna, P.; de Arquer, F. P. G.; Zhang, B.; Becknell, N.; Ross, M. B.; Li, Y.; Banis, M. N.; Li, Y.; Liu, M. J. J., Sulfur-modulated tin sites enable highly selective

electrochemical reduction of CO_2 to formate. **2017,** *1* (4), 794-805.
16. Li, F.; Chen, L.; Xue, M.; Williams, T.; Zhang, Y.; MacFarlane, D. R.; Zhang, J., Towards a better Sn: Efficient electrocatalytic reduction of CO_2 to formate by Sn/SnS_2 derived from SnS_2 nanosheets. *Nano Energy* **2017,** *31*, 270-277.
17. Tian, J.; Wang, R.; Shen, M.; Ma, X.; Yao, H.; Hua, Z.; Zhang, L., Bi–Sn Oxides for Highly Selective CO_2 Electroreduction to Formate in a Wide Potential Window. *ChemSusChem n/a* (n/a).
18. Wen, G.; Lee, D. U.; Ren, B.; Hassan, F. M.; Jiang, G.; Cano, Z. P.; Gostick, J.; Croiset, E.; Bai, Z.; Yang, L.; Chen, Z., Orbital Interactions in Bi-Sn Bimetallic Electrocatalysts for Highly Selective Electrochemical CO_2 Reduction toward Formate Production. **2018,** *8* (31), 1802427.
19. Li, Z.; Cao, A.; Zheng, Q.; Fu, Y.; Wang, T.; Arul, K. T.; Chen, J.-L.; Yang, B.; Adli, N. M.; Lei, L.; Dong, C.-L.; Xiao, J.; Wu, G.; Hou, Y., Elucidation of the Synergistic Effect of Dopants and Vacancies on Promoted Selectivity for CO_2 Electroreduction to Formate. *Advanced Materials* **2021,** *33* (2), 2005113.
